# Black Caregivers’ Perceptions of Quality of Care and the Role of Advance Care Planning Discussions

**DOI:** 10.1093/geroni/igaf122.2604

**Published:** 2025-12-31

**Authors:** Jessica Yauk, Debra Dobbs, Lindsay Peterson

**Affiliations:** University of South Florida, Tampa, Florida, United States; University of South Florida, Tampa, Florida, United States; University of South Florida, Tampa, Florida, United States

## Abstract

There are distinct variations in patterns of healthcare quality between minority and non-minority older adults. Black family caregivers are at the crux of the care convoy for Black older adults needing ongoing care. This study sought to understand informal Black caregivers’ perceptions of care quality across the care continuum, with a specific focus on advance care planning (ACP). The National Health and Aging Trends Study last-month-of-life interview and the National Consensus Project Practice Guidelines for Quality Palliative Care framework guided the interview questions used in this study. Seven Black caregivers of seriously ill older adults were recruited in Florida. Caregivers completed one interview describing interactions with healthcare professionals and their perceptions of care quality and ACP engagement. The results identified themes including communication, care transitions, culturally relevant care, and the gaps in health literacy. Caregivers expressed a need for better communication from providers regarding diagnosis and disease trajectory and a need for ongoing relationships with care recipients to understand care needs. Care transitions were perceived as high-quality when there was clear communication between providers and family. In contrast, caregivers felt less confident in decision-making when they thought they did not have all the information or resources required to care for the care recipient. Regarding ACP, caregivers indicated that ACP was an important aspect of goal-concordant care. Practice implications to improve health care quality for Black older adults include better communication, culturally competent care and more effective care coordination. Challenges in recruiting older Black caregivers will also be addressed.

